# Phenotypic Characterization of Encephalitis in the BRAINS of Badgers Naturally Infected with Canine Distemper Virus

**DOI:** 10.3390/ani13213360

**Published:** 2023-10-29

**Authors:** Israel Espinoza, María José García Iglesias, Álvaro Oleaga, María Gracia de Garnica García, Ana Balseiro

**Affiliations:** 1 Departamento de Sanidad Animal, Universidad de León, 24071 León, Spain; iespih00@estudiantes.unileon.es (I.E.); mjgari@unileon.es (M.J.G.I.); mgarng00@estudiantes.unileon.es (M.G.d.G.G.); 2 Instituto Universitario (LOU) de Biomedicina (IBIOMED), Universidad de Léon, 24071 León, Spain; 3 Sociedad de Servicios del Principado de Asturias S.A. (SERPA), 33203 Gijón, Spain; alvaroleaga@yahoo.es; 4 Micros Veterinaria, S.L., 24007 León, Spain; 5 Departamento de Sanidad Animal, Instituto de Ganadería de Montaña (CSIC—Universidad de León), 24346 León, Spain

**Keywords:** canine distemper virus, badger *Meles meles*, local immune response, immunohistochemistry, brain

## Abstract

**Simple Summary:**

The local immune response within the central nervous systems (CNSs) of seven badgers naturally infected with canine distemper virus (CDV) in Asturias (Atlantic Spain) was evaluated via immunohistochemistry. Microglia and astrocytes were the cell types present in the foci of gliosis, whereas T or B lymphocytes were absent. The knowledge gained in the field of the immunopathogenesis of diseases affecting the CNS could clarify CDV disease patterns in badgers.

**Abstract:**

Canine distemper virus (CDV) affects a huge diversity of domestic and wild carnivores, with increasing numbers of mortality events worldwide. The local cell-mediated immune response elicited against a natural infection is an important factor in determining the outcome of CDV infection. Therefore, the purposes of this study were to describe the local immune response within the central nervous systems (CNSs) of seven badgers naturally infected with CDV in Asturias (Atlantic Spain) and to determine the phenotype and distribution of microglial cells, T and B lymphocytes, and astrocytes in the foci of gliosis located in the thalamus and cerebellum using immunohistochemistry. The immunohistochemical assessment demonstrated the presence of Iba1-positive microglia and GFAP-positive astrocytes in the foci of gliosis, whereas T (CD3-negative) or B (CD20-negative) lymphocytes in those same lesions were absent. Our results also revealed that the badgers with natural CDV encephalitis presented lesions mostly located in the white matter of the thalamus and cerebellum, suggesting a CDV-specific tropism for the white matter of badger brains in those locations. The knowledge gained in the field of the immunopathogenesis of distemper disease affecting the CNSs of badgers could help to clarify CDV disease patterns in this species.

## 1. Introduction

**Figure 1:**
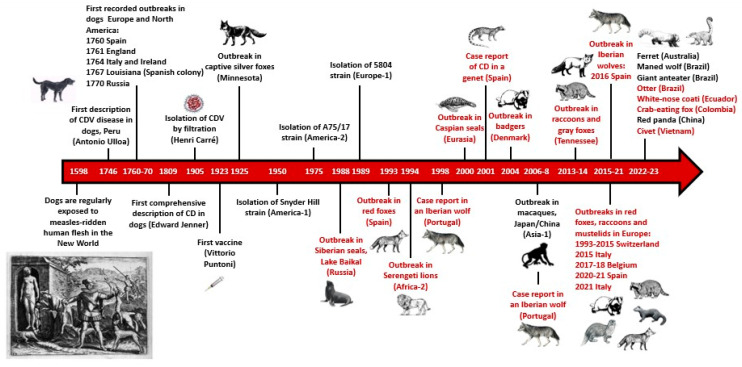
The main historical events related to canine distemper virus (CDV) since its discovery in the 16th century. The letters in red color refer to outbreaks or cases of wildlife. CD: distemper disease. The engraving showing dogs feeding on human remains corresponds to Plate XIII of the Narratio regionum indicarum per Hispanos quosdam devastatarum verissima [4]. Data source: Scopus. Author: A. Balseiro.

Infectious diseases are one of the most important causes of population mortality in wild mammal carnivore populations all around the world [1]. Among them, viral diseases that affect both wild and domestic animals are continuously emerging and re-emerging due to human interference and environmental factors, such as climate change, livestock and agricultural practices, international trade, and urbanization [1]. This relates to viruses included in the family *Paramixoviridae*, which affect a wide variety of vertebrates, from fish to mammals. This family comprises four genera—*Morbillivirus*, *Rubulavirus*, *Respirovirus*, and *Henipavirus*—which spread primarily via the respiratory route [2]. Historically, the genus *Morbillivirus* has caused catastrophic outbreaks among humans and animals. The single-stranded RNA viruses included in this genus are human measles virus, bovine rinderpest virus, and canine distemper virus (CDV) [2]. Indeed, human measles virus breached the species barrier in South America in the 16th century due to dogs having access to the remains of people who died from measles [3,4] (Figure 1). CDV is the causative agent of distemper and has a high pathogenic capacity [5]. This virus affects domestic and wild animals worldwide, especially carnivores [6]. Recently, numerous high-mortality distemper outbreaks have occurred throughout the world among canids, felids, ursids, mustelids, and even non-human primates [5,7,8,9,10,11,12,13,14] (Figure 1). Among the mustelids, badgers (*Meles meles*) have been diagnosed with distemper in several studies [13,15], and further investigations are requested to assess the role of badgers in the epidemiology and evolution of distemper in different countries [13,16].

CDV produces similar lesions in wildlife, including badgers and dogs [13,17]. The main routes of infection are oral or nasal. Once the virus comes into contact with the tonsils and the upper respiratory tract, it starts multiplying within the macrophages [18]. After that, viremia starts with the spreading of the CDV along the lymphatic system to the lymphoid organs, the gastrointestinal tract, the liver, the kidneys, and the central nervous system (CNS). Specifically, CDV reaches the brain through infected mononuclear cells that across the blood–brain barrier, and via circulation in the cerebrospinal fluid, which is associated with ependyma that coats the ventricles [13,19]. The infection causes histologic lesions and intracytoplasmic and intranuclear inclusion bodies in any of the affected tissues [5].

The pathogenesis of distemper in the CNS is the result of complex interactions between CDV and its host. In acute viral encephalitis, the recruitment of immune cells into the CNS plays a fundamental role in the outcome of the disease [20]. The immune response in the CNS against CDV infection has been described not only in naturally and experimentally infected dogs [21,22], but also in ferrets [23,24], and in red foxes with spontaneous CDV encephalitis as well [12]. However, little is known about the local immune response in the brains of badgers naturally infected with CDV.

In 2020, an outbreak of distemper occurred in carnivores, including free-ranging Eurasian badgers, in Asturias (Atlantic coast of Spain) [13]. The most frequent clinical signs were ataxia, tremors or seizures, vision alteration, and ocular and nasal discharges associated with conjunctivitis and rhinitis, respectively. The pathological examination revealed lesions mainly in the lungs (i.e., interstitial pneumonia), CNS (i.e., non-purulent meningoencephalomyelitis involving the following types of damage in various areas of the brain, mostly thalamus and cerebellum: meningitis, neuronophagia, neuronal degeneration and necrosis, demyelination, and multifocal gliosis), and lymph nodes and spleen (i.e., lymphoid depletion). Immunohistochemistry against CDV revealed that the virus was mainly present in the corpus striatum, thalamus, and hypothalamus, rather than in the other regions of the brain [13]; however, the local immune response in the CNS was not evaluated.

The purposes of this study were (i) to describe the local immune response within the CNSs of badgers naturally infected with CDV and, (ii) to determine the phenotype and distribution of microglial cells, T and B lymphocytes and astrocytes in the foci of gliosis.

## 2. Materials and Methods

### 2.1. Badgers

Seven badgers identified from 1 to 7 were used in this study. The animals died due to clinical distemper disease during the outbreak that occurred in Asturias (northern Spain) in 2020 [13]. All were diagnosed with distemper via qPCR, and their histological lesions have been described in [13]. The thalamus and cerebellum were identified as the brain regions showing the most severe lesions; therefore, we chose both locations for local cell immune response assessment in the present study.

### 2.2. Immunohistochemistry

For each badger, serial paraffin-embedded sections (3 µm) were used for the immunohistochemical identification of four cell types using the following commercial monoclonal or polyclonal primary antibodies (Table 1): ionized calcium-binding adaptor molecule 1 (Iba1) for microglial cells, CD3 for T lymphocytes, CD20 for B lymphocytes, and glial fibrillary acidic protein (GFAP) for astrocytes. Firstly, slices were deparaffinized using xylene and alcohol and, afterwards, they were rehydrated, and antigens were retrieved with sodium citrate buffer (10 mmol/L, pH 6.0) by heat induction using a microwave for 20 min. Then, after blocking endogenous peroxidase activity via incubation in a hydrogen peroxide (0.5%) solution in distilled water for 30 min at room temperature, the nervous tissue sections were incubated overnight at 4 °C in a humidified chamber with commercial monoclonal or polyclonal antibodies diluted in TBS + BSA 0.1% (Table 1). The following day, the slices were washed with TBS 1×, and incubated with horse anti-mousse or goat anti-rabbit secondary antibody (Vector Laboratories, Newark, CA, USA) diluted at 1:200 in TBS + BSA 0.1% (Table 1). Afterwards, incubation with the Avidin–biotin–peroxidase complex reagent method (ABC Standard, Vector Laboratories, CA, USA) in TBS 1× for 30 min was carried out, and labeling was visualized via the application of the Vector^®^ NovaRed™ peroxidase substrate kit (Vector Laboratories, CA, USA) as a chromogen substrate. Finally, slices were counterstained with Mayer’s haematoxylin for 45 s, dehydrated, and mounted with DPX (Fluka, Sigma, St. Louis, MO, USA). The negative control consisted of an additional slice without the primary antibody. Lymph node tissue from a healthy badger was used as a positive control for Iba1, CD3, and CD20 antibodies. Samples of the CNS of a healthy badger were used as the positive control for the GFAP antibody.

### 2.3. Evaluation and Quantification of Cellular Types

Immunostained tissue sections of the badgers´ thalamus and cerebellum were scanned at the Microscopy Service of the University of León. An Olympus BX51 microscope (Olympus, Tokyo, Japan) and an Olympus XC10 camera (Olympus, Tokyo, Japan) were used, and the digital images were visualized using OlyVIA version 2.9 software (Olympus Münster, Germany). The virtual images were classified according to the CNS region (thalamus or cerebellum), location (white or gray matter), and cell type (microglia, T and B lymphocytes, and astrocytes). The percentage of positively immunolabelled area (μm^2^) in five microscopic fields at 200× magnification showing lesions in the thalamus and cerebellum was quantified for each image of Iba1- and GFAP-labelled microglial cells and astrocytes, respectively. The area with an immunohistochemical-positive reaction was determined using the Nikon NIS-Elements 3.20 image analysis software (Imaging Software 3.20, Nikon Instruments Inc., Cambridge, MA, USA) after setting the thresholds. The results are expressed as the proportion of the positively immunolabelled area within the total area of the selected site. The number of immunolabelled foci of gliosis in those fields was also counted. No immunolabelling of T and B lymphocytes was observed, and therefore, their quantification was not applicable.

### 2.4. Statistical Analysis

The data obtained on the percentage of immunolabelled areas in the thalamus and cerebellum of badgers for each marker were analyzed using SPSS software version 26.0 for Windows. The comparison of the percentages of immunohistochemical expression of Iba1 and GFAP according to their quantity, location in the thalamus and cerebellum, and location in the white or gray matter was performed. In addition, the comparison of the immunolabelling of both markers at the same location was performed. The normality assumption for these quantitative variables was assessed using the Shapiro–Wilk test (*n* < 50). A parametric Student´s *t*-test or non-parametric Mann–Whitney U test for two samples was applied according to whether the variables were normally distributed or not, respectively. To compare groups in the percentage of immunostaining with the Iba1 antibody (a variable that did not follow a normal distribution), the non-parametric Mann–Whitney U test for two samples was used. The percentage of GFAP expression (a variable that followed a normal distribution) was analyzed using the parametric Student’s *t*-test. The equality of variances was also assessed using Levene’s test. The results are expressed as mean ± SD (standard deviation), median, and interquartile range. For all the statistics, we considered a *p*-value < 0.05 as significant.

## 3. Results

### 3.1. Cell types in Foci of Gliosis

**Figure 2:**
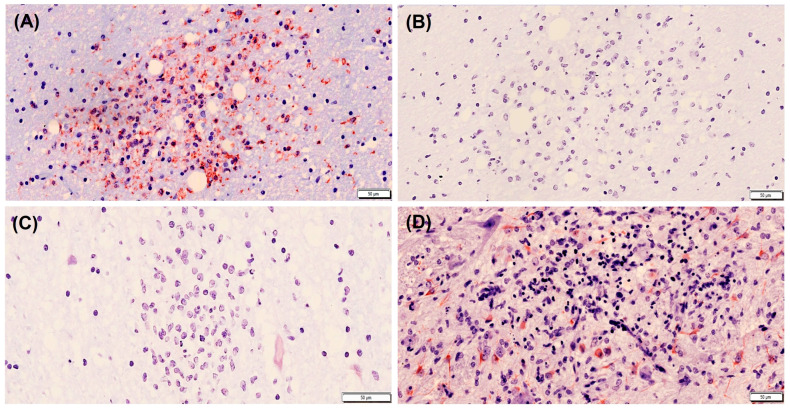
Cell types present in a focus of gliosis in the thalamus of a badger naturally infected with canine distemper virus. Immunolabelling against microglia (**A**), T lymphocytes (**B**), B lymphocytes (**C**), and astrocytes (**D**). Note the absence of T and B lymphocytes. Avidin–biotin–peroxidase complex. Contrast of nuclei with hematoxylin. Primary antibodies: ionized calcium-binding adaptor molecule 1 (Iba1) for microglial cells, CD3 for T lymphocytes, CD20 for B lymphocytes and glial fibrillary acidic protein (GFAP) for astrocytes. Scale bar = 50 microns. Note that these are representative images.

The immunohistochemical assessment of the cellular immune response in the brains of seven badgers naturally infected with CDV demonstrated the presence of Iba1-positive microglia and GFAP-positive astrocytes in the foci of gliosis, whereas T or B lymphocytes in those same lesions were absent (Figure 2). The statistical analysis of the data obtained in the quantification of immunolabelling of microglia and astrocytes at two locations in the brain (thalamus and cerebellum) showed that the Iba1-positive microglia activation level was significantly higher than that of the astrogliosis (GFAP-positive) in the cerebellum of the animals examined (Table 2). The same immunolabelling pattern was observed in the thalamus with a clear trend towards significance (Table 2).

### 3.2. Comparison of Activation of Microglia in the Thalamus and Cerebellum

**Figure 3:**
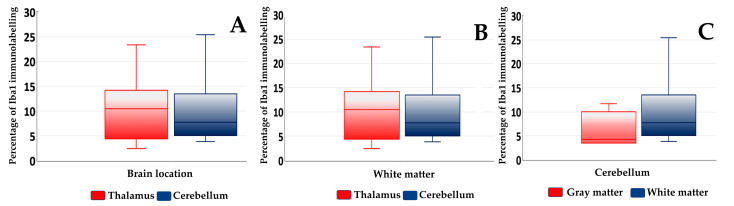
Box plots of median and interquartile range of percentage of Iba1 immunolabelling in foci of gliosis of the thalamus and cerebellum of badgers naturally infected with canine distemper virus. Comparisons of Iba1 expression between the two locations of the brain analyzed (**A**) between the white matter of the thalamus and cerebellum (**B**) and between the gray and white matters of the cerebellum are shown (**C**).

**Figure 4:**
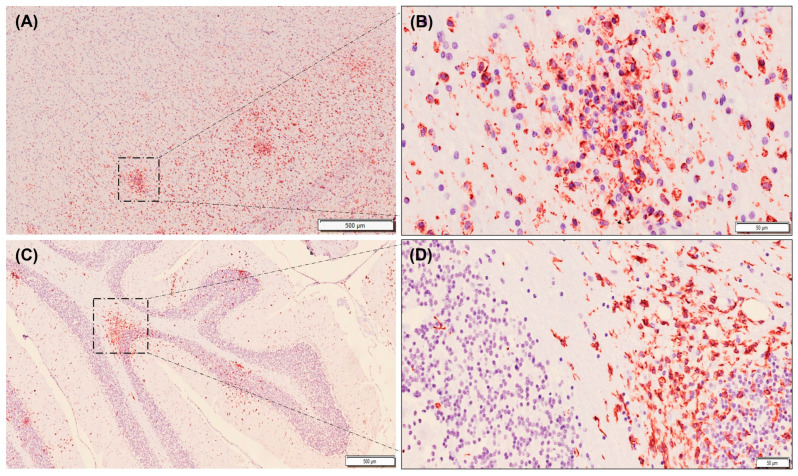
Immunolabelling of microglia using Iba1 antibody (stained red) in tissue sections from the thalamus (**A**,**B**) and cerebellum (**C**,**D**) of badgers naturally infected with canine distemper virus. Note that immunolabelling is slightly higher in thalamus (**A**) than in cerebellum (**C**). Also note that in the cerebellum, positive immunolabelling is higher in white matter than in gray matter (**C**,**D**). Avidin–biotin–peroxidase complex. Contrast of nuclei with hematoxylin. Scale bar = 500 microns (**A**,**C**) and 50 microns (**B**,**D**). Note that these are representative images.

The immunolabelling with Iba1 (Table 3) was found to be higher in the foci of gliosis in the thalamus than in those located in the cerebellum, although this difference was not statistically significant (Figure 3 and Figure 4). When the Iba1 expression levels in the white matter in both brain locations were assessed, they were found to be similar in the thalamus and cerebellum, with no significant differences between both locations. When comparing the percentage of Iba1-positive microglia in focal gliosis located in the white and gray matters of the cerebellum, a higher expression level was observed in the white matter than in the gray one, although no statistically significant difference could be demonstrated (Figure 3).

### 3.3. Comparison of Astrogliosis in the Thalamus and Cerebellum

**Figure 5:**
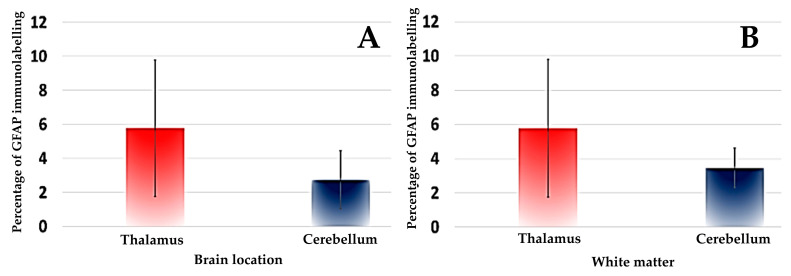
Box plots of median and interquartile range of percentage of GFAP immunolabelling in foci of gliosis of the thalamus and cerebellum of badgers naturally infected with canine distemper virus. Comparisons of GFAP expression between the two locations of the brain analyzed (**A**) and between the white matter of the thalamus and cerebellum are shown (**B**).

**Figure 6:**
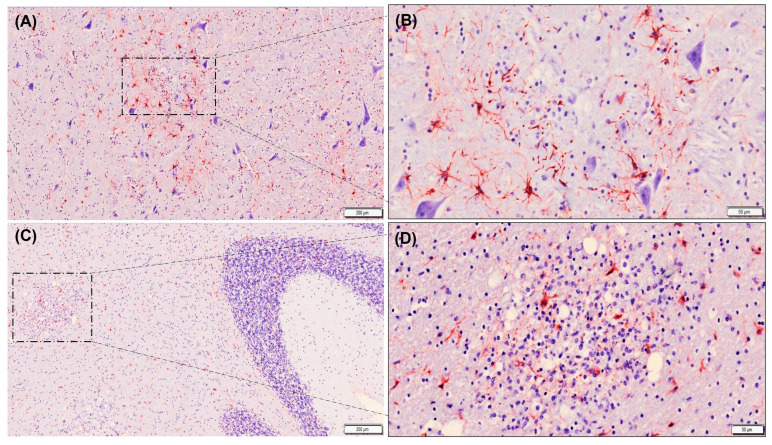
Immunolabelling of astrocytes using GFAP antibody (stained red) in tissue sections from thalamus (**A**,**B**) and cerebellum (**C**,**D**) of badgers naturally infected with canine distemper virus. Note that GFAP expression level is slightly higher in foci of gliosis in the thalamus (**A**,**B**) compared to those located in white matter of the cerebellum (**C**,**D**). Note also that less astrogliosis is observed in gray matter in relation to white matter in the cerebellum (**C**). Avidin–biotin–peroxidase complex. Contrast of nuclei with hematoxylin. Scale bar = 200 microns (**A**,**C**) and 50 microns (**B**,**D**). Note that these are representative images.

The statistical study showed that the mean GFAP expression level was higher in the foci of gliosis in the thalamus compared to those located in the cerebellum, although no significant differences were found (Table 4; Figure 5 and Figure 6). A similar pattern of expression was observed when the GFAP-positive astrocytes were assessed only in the white matter of the cerebellum and thalamus (Table 4; Figure 5 and Figure 6). On the other hand, less astrogliosis in the cerebellum was observed in the gray matter in relation to the white one (0.61% versus 3.46%, respectively), although no statistical comparison was carried out due to insufficient data in the gray matter.

## 4. Discussion

This study describes the local immune response in the CNSs of badgers naturally infected with the CDV. In all the badgers examined, the microglia proved to be the most abundant cell type in the areas of CNS injury. This finding was previously reported by some other authors in CDV infections in wild carnivores and domestic dogs [12,25,26]. The increase in this cell type may be associated with the fact that microglia is the main effector element of the immune response in the CNS to any neuropathological event, as mediated through signals received by the cytokines [27]. In response to an injury or pathogen invasion, the microglia become activated [28], migrate, and accumulate at the site of injury through a process known as chemotaxis [29]. Once activated, they are involved in several functions aimed at eliminating the pathogen or treating the injury, such as the release of proinflammatory molecules, increased phagocytic capacity, antigen presentation, attraction of T lymphocytes, and tissue repair [30,31,32,33,34]. Furthermore, some studies show that in CDV infections, there is a clear association between microglia activation and the pathogenesis of demyelination [25].

On the other hand, astrocytes, which play a significant role in the formation of the blood–brain barrier [35], were the other cell type that reacted to a natural infection with CDV in all the badgers, although they were found in a lower quantity than that of the microglia. Several studies in wild carnivores and domestic dogs concluded that the astrocytes were the main cell population infected by CDV [10,12,21,24,36,37,38,39] at the early stages of CDV infection [40]. These cells, once infected with CDV, react with changes, including hypertrophy, hyperplasia, and changes in their protein expression profile [19,36,41,42,43]. Astrocytes may also contribute to early demyelination by releasing proinflammatory cytokines and myelinotoxic factors [26,44,45]. In that regard, previous studies showed that CNS demyelination is associated with the increased expression of interleukin IL-1, IL-12, tumor necrosis factor (TNF-α), and transforming growth factor (TGF-β) [44]. TNF-α is highly expressed in astrocytes and seems to play a crucial role in the pathogenesis of early demyelination [44]. Thus, the initial production of TNF-α would establish a “vicious” cycle of attracting inflammatory cells (i.e., lymphocytes) to CNS lesions, contributing to the synthesis of more cytokines and the development of the chronic stage of demyelinating leukoencephalitis [26,44,46].

An unexpected finding in this study was the absence of T and B lymphocytes in all the tissue sections examined from the badgers´ thalamus and cerebellum. In contrast, the invasion of T cells in the CNS elicited by the microglia and astrocyte activation has been described in dogs [22,45]. Our results could be due to the high immunosuppressive capacity of CDV demonstrated in natural and experimental infections, which is related to the severity of the disease and the persistence of this pathogen in the lymphoid tissue and CNS [12,26]. Previous studies in canids and ferrets experimentally infected with CDV showed that the animals developed an inhibited cellular immune response as well as the decreased proliferation of T and B lymphocytes [25,47,48,49,50,51]. In CDV infection, acute-phase lymphopenia is characterized by a depletion of T-helper (CD4+), cytotoxic T (CD8+), and B (CD21+) cells in the peripheral blood [26,46]. The impairment of lymphoid tissues by virus infection may therefore explain the low-level production of those immune cell types. Furthermore, different studies demonstrated programmed cell death (“apoptosis”) in many non-infected lymphocytes, which would indicate the existence of virus-independent apoptotic mechanisms [52,53], such as the overactivation of the innate immune system or apoptosis induced by FAS-mediated activation or the “death receptor” (membrane glycoprotein) of lymphoid cells [54,55]. Another theory is that CDV modulates monocyte functions, with a resulting inhibition of interleukin IL-1 and increased prostaglandin E2 release, impairing antigens’ presentation via monocytes, and therefore, contributing to a decreased immune response due to the severe negative effect on B-lymphocyte differentiation, plasma cell formation, and immunoglobulin production [26]. On the other hand, the virus N protein acts indirectly on T-lymphocyte function by modulating dendritic cell antigen presentation, thereby compromising FCγ receptor (CD32)-expressing cells, and causing the suppression of non-infected cells, with a resulting decrease in the level of interleukin IL-12, in a very similar way to what has been described in measles virus disease [56]. The absence of an effective humoral response would lead to an acute clinical status, usually a fatal one [26]. Finally, the clinical stage of badgers must also be considered, since all the animals included in this study succumbed to distemper. In that regard, only the dogs experimentally infected with CDV that recovered from distemper showed high activity of antiviral immunity mediated by lymphocytes, whereas those that died due to distemper had little or no response [57]. Therefore, it is possible that during subclinical infections or at the early stages of infection [45], lymphocytes might be present in the CNSs of CDV-infected badgers. Further studies must be performed to confirm this hypothesis.

CDV causes multifocal lesions in the gray and white matters; however, our results revealed that the badgers with natural CDV encephalitis present lesions mostly located in the white matter in the thalamus and cerebellum, suggesting a CDV-specific tropism for the white matter of badger brains, affecting mainly those regions. In contrast, in dogs, preferential sites are the cerebellum and periventricular white matter, especially around the fourth ventricle, optical pathways, and spinal cord [19].

Finally, Figure 1 reflects the continuous spreading of distemper in wildlife worldwide, even affecting endangered species. Moreover, human-populated areas with overlapping wild habitats facilitate interspecies interactions (i.e., dogs and wild carnivores), thus raising the opportunities of disease transmission [13]. These issues are factors that generate a breeding ground that increases the probabilities of viral genetic mutations that may lead to changes in the pathogenesis, virulence, specificity, and efficacy of current commercial vaccines, as well as to the rapid spreading and enlargement of the host spectrum in new outbreaks [8]. In this scenario, the knowledge gained in the field of the immunopathogenesis of distemper in its wildlife hosts is crucial as a basis for establishing future control measures, and therefore minimizing distemper spillover and its consequences for wild carnivore populations in an effort to preserve biodiversity and animal health.

## 5. Conclusions

This study contributes to the progress made in understanding badgers’ local immune response to CDV infection in CNS. In badgers with naturally acquired CDV encephalitis, the intrinsic cell response based on the microglia and astrocytes suggests a non-specific innate immune response, primarily mediated by the microglia. However, the extrinsic immune response, predominantly based on T and B lymphocytes, was absent, supporting the immunosuppressive capacity of CDV in badgers. The local immune response against natural CDV infection is relevant in determining the outcome of the infection [45]. In this regard, the cell-mediated immune response may contribute to nervous tissue damage, mainly based in the releasing of proinflammatory cytokines which, in some cases, can lead to a fatal outcome, as was shown in the badgers in this study.

## Figures and Tables

**Table 1:** Primary and secondary antibodies used for cell-type characterization.

Primary Antibody (Dilution)	Cell Type Detected	Clone No.	Source	Secondary Antibody (Dilution)
Iba1 ^1^ (1:1000)	Microglia	Polyclonal 019-19741	FLUJIFILM-Wako Chemicals Eu-rope GmbH, Neuss, Germany	Goat anti-rabbit (1:200)
CD3 (1:500)	T lymphocytes	Monoclonal NCL-L-CD3-565	Novacastra, Leica Biosystem, Newcastle, UK	Horse anti-mouse (1:200)
CD20 (1:400)	B lymphocytes	Polyclonal PA5-16701	ThermoFisher, MA, USA	Goat anti-rabbit (1:200)
GFAP ^2^ (1:200)	Astrocytes	Monoclonal MCA-5C10-AP	EncorBiotechnology, Gainesville, FL, USA	Horse anti-mouse (1:200)

^1^ Ionized calcium-binding adapter molecule 1. ^2^ Glial fibrillary acidic protein.

**Table 2:** Comparison between Iba1-positive microglia and GFAP-positive astrocytes in foci of gliosis located in the thalamus and cerebellum of badgers naturally infected with canine distemper virus.

Brain	Marker	Number of Foci of Gliosis *	Mean ± SD **	Median (Interquartile Range)	*p* Value
Thalamus					0.059
	Iba1	14	10.55 ± 6.38	10.52 (4.30–14.52)	
	GFAP	9	5.78 ± 4.01	5.29 (2.95–6.74)	
Cerebellum					0.008
	Iba1 GFAP	14 4	8.98 ± 6.24 2.75 ± 1.71	6.54 (4.67–12.37) 2.81 (1.57–3.92)	

* Iba1-positive or GFAP-positive foci of gliosis evaluated in five fields at 200× magnification. ** SD, standard deviation. Results are expressed as percentage of Iba1 or GFAP immunolabelling in 5 fields at 200×. Mann–Whitney U test for Iba1 or Student´s *t*-test for GFAP was applied.

**Table 3:** Iba1-expressing microglia in foci of gliosis located in the thalamus and cerebellum of badgers naturally infected with canine distemper virus.

Brain	Number of Foci of Gliosis *	Mean ± SD **	Median (Interquartile Range)	*p* Value
**Location** Thalamus Cerebellum				0.60
	14	10.55 ± 6.38	10.52 (4.30–14.52)	
	14	8.98 ± 6.24	6.54 (4.67–12.37)	
**White matter** Thalamus Cerebellum				1.00
	14 10	10.55 ± 6.38 10.19 ± 6.74	10.52 (4.30–14.52) 7.77 (4.76–13.86)	
**Cerebellum** Gray matter White matter				0.14
	4 10	5.95 ± 3.92 10.19 ± 6.74	4.29 (3.51–8.38) 7.77 (4.76–13.86)	

* Iba1-positive foci of gliosis evaluated in five fields at 200× magnification. ** SD, standard deviation. Results are expressed as percentage of Iba1 immunolabelling in five fields at 200×. Mann–Whitney U test was applied.

**Table 4:** Glial fibrillary acidic protein (GFAP)-expressing astrocytes in foci of gliosis located in the thalamus and cerebellum of badgers naturally infected with canine distemper virus.

Brain	Number of Foci of Gliosis *	Mean ± SD **	Median (Interquartile Range)	*p* Value
**Location** Thalamus Cerebellum				0.26
	9	5.78 ± 4.01	5.29 (2.95–6.74)	
	4	2.75 ± 1.71	2.81 (1.57–3.92)	
**White matter** Thalamus Cerebellum				0.60
	9 3	5.78 ± 4.01 3.46 ± 1.15	5.29 (2.95–6.74) 3.09 (2.81–3.92)	

* GFAP-positive foci of gliosis evaluated in five fields at 200× magnification. ** SD, standard deviation. Results are expressed as percentage of GFAP immunolabelling in five fields at 200×. Student’s *t* test was applied.

## Data Availability

The data that support the findings of this study are available from the corresponding author upon reasonable request.
